# The role of cardiac computed tomography in predicting adverse coronary events

**DOI:** 10.3389/fcvm.2022.920119

**Published:** 2022-07-15

**Authors:** Maria Emfietzoglou, Michail C. Mavrogiannis, Athanasios Samaras, Georgios P. Rampidis, George Giannakoulas, Polydoros N. Kampaktsis

**Affiliations:** ^1^Division of Cardiovascular Medicine, Radcliffe Department of Medicine, University of Oxford, John Radcliffe Hospital, Oxford, United Kingdom; ^2^Aristotle University of Thessaloniki, Thessaloniki, Greece; ^3^Division of Cardiology, Columbia University Irving Medical Center, New York, NY, United States

**Keywords:** cardiac computed tomography, coronary artery disease, coronary artery calcium score, perivascular fat, computational fluid dynamics, adverse coronary events

## Abstract

Cardiac computed tomography (CCT) is now considered a first-line diagnostic test for suspected coronary artery disease (CAD) providing a non-invasive, qualitative, and quantitative assessment of the coronary arteries and pericoronary regions. CCT assesses vascular calcification and coronary lumen narrowing, measures total plaque burden, identifies plaque composition and high-risk plaque features and can even assist with hemodynamic evaluation of coronary lesions. Recent research focuses on computing coronary endothelial shear stress, a potent modulator in the development and progression of atherosclerosis, as well as differentiating an inflammatory from a non-inflammatory pericoronary artery environment using the simple measurement of pericoronary fat attenuation index. In the present review, we discuss the role of the above in the diagnosis of coronary atherosclerosis and the prediction of adverse cardiovascular events. Additionally, we review the current limitations of cardiac computed tomography as an imaging modality and highlight how rapid technological advancements can boost its capacity in predicting cardiovascular risk and guiding clinical decision-making.

## Introduction

**Figure 1:**
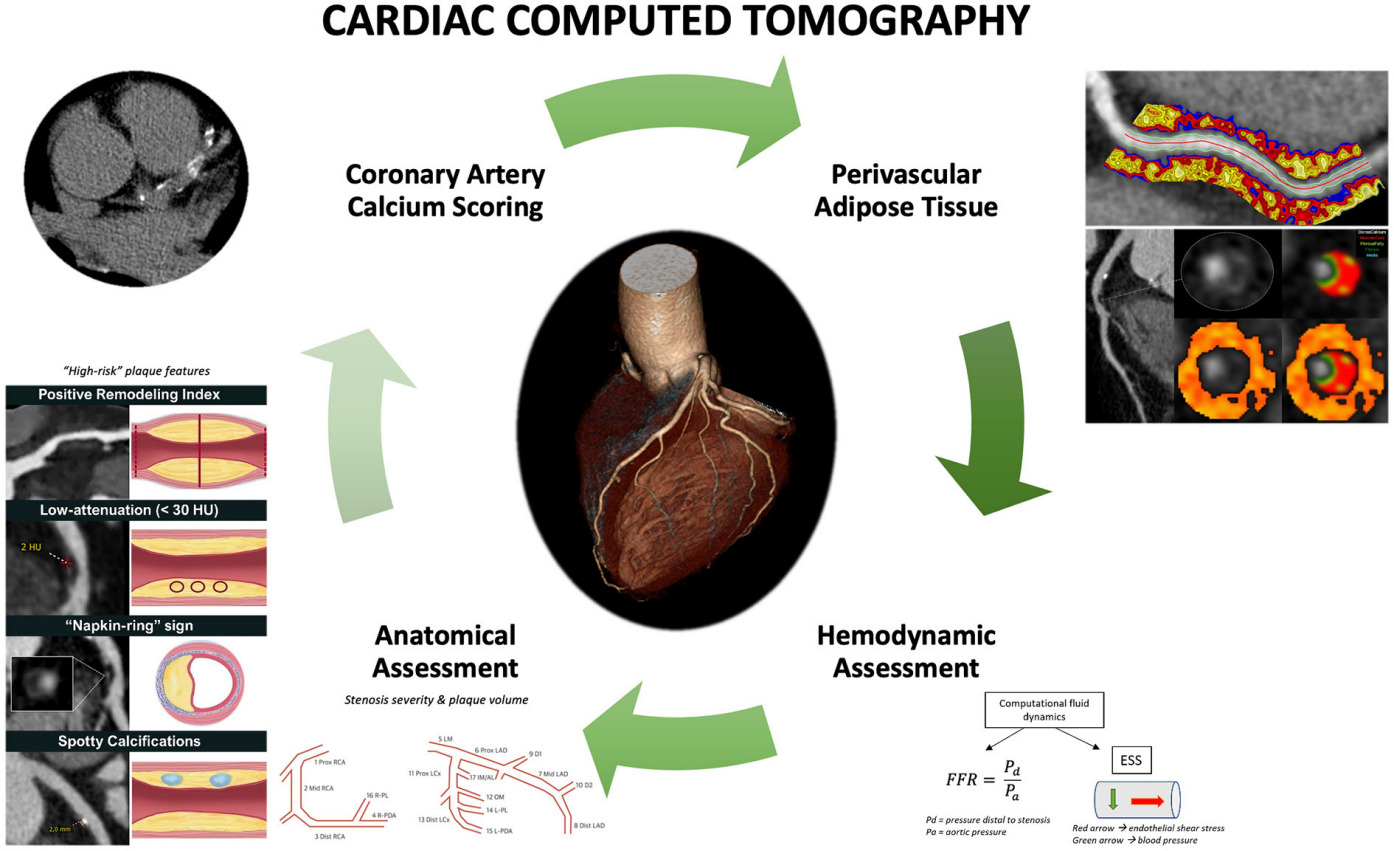
Current and emerging roles of cardiac computed tomography in predicting adverse coronary events. Comprehensive assessment of coronary artery disease with cardiac computed tomography (CCT) includes coronary artery calcium score, anatomic assessment to identify stenosis, plaque volume, and high-risk plaque features, hemodynamic assessment using computational fluid dynamics to compute fractional flow reserve (FFR) and endothelial shear stress (ESS), as well as derivation of perivascular fat attenuation index. Perivascular fat attenuation index figure is owned by: Oxford Academic Cardiovascular CT Core Lab and Lab of Inflammation and Cardiometabolic Diseases at NHLBI, published under Attribution-NonCommercial 2.0 Generic (CC BY-NC 2.0). Link to license: https://creativecommons.org/licenses/by-nc/2.0/.

Cardiac computed tomography (CCT) has emerged in the last decade as an important non-invasive modality for the evaluation of coronary artery disease (CAD) with actively expanding indications. Initial research led to the establishment of a coronary artery calcium score (CACS) for improved risk stratification of asymptomatic patients with intermittent probability for adverse atherosclerotic events; a CACS of zero is associated with low rates of future adverse events (1). Similarly, CCT angiography (CCTA) was shown to be a reliable modality for ruling out CAD in low-risk patients who present to the emergency room with chest pain, leading additionally to decreased length of stay (2). Further studies examined the role of cardiac CCTA in the evaluation of suspected CAD in patients with stable angina. The non-invasive anatomic assessment was compared to standard of care non-invasive functional assessment as an additional or standalone modality (3, 4). The addition of CCTA resulted in a lower risk for long-term coronary death or myocardial infarction, as the increased sensitivity of CCTA for the detection of coronary atherosclerosis led to higher rates of guideline-directed preventive therapy initiation. The use of CCTA as a standalone modality resulted in similar outcomes compared to stress testing or invasive coronary angiography (5). These results highlighted that CCTA is an excellent non-invasive modality for the evaluation of suspected CAD in symptomatic patients; in fact, the latest European Society of Cardiology (ESC) guidelines give a class I recommendation for cardiac CCTA for the evaluation of stable CAD (6). Additionally, however, these results underline the importance of complementary functional assessment that so far had been obtained by stress testing and invasive indices. The latter represents an active area of research in CCTA that could improve its prognostic value for coronary events in patients where coronary atherosclerosis is diagnosed (7, 8). Parallel to the above, a significant number of studies focused on the potential role of CCTA for the identification of “vulnerable plaques”, i.e., coronary atherosclerosis sites that would be associated with a much higher chance of plaque rupture and thrombotic events (9). Although today, the focus has switched to the “vulnerable patient” (10), assessment of high-risk plaques remains important. Finally, CCTA has recently been pivotal in understanding the role of perivascular adipose tissue in atherosclerosis (11) and how associated pericoronary inflammation can be used as a novel prognostic index of adverse coronary outcomes (12) (Figure 1).

In the current review, we aim to delineate the contribution of CCT in predicting future adverse cardiovascular outcomes across different clinical scenarios. First, we discuss how CCT can quantitatively assess coronary calcium, the degree of luminal stenosis and total plaque burden, and how these are related to adverse events. We then discuss the role of CCTA based-plaque characterization and its clinical role. Furthermore, we summarize the current state of functional and hemodynamic assessment *via* CCTA including endothelial shear stress. We elaborate on the role of perivascular fat inflammation in relation to CCTA. Finally, we appraise the limitations of CCT and discuss how technological advances can enhance the capability of CCT in cardiovascular risk prediction in the near future.

## Coronary artery calcium score

CCT allows quantification of plaque calcium burden by measuring the Agatston score (13). CACS is based on a low radiation dose, non-contrast CT (14) and represents a simple, quick, inexpensive, and reproducible test. CACS has shown to correlate well with long-term risk of cardiac events when used as a binary or categorical number (1, 15–18). For example, the 10-year risk for adverse atherosclerotic events of a 65-year-old male with hyperlipidemia and medically treated hypertension is over 10%. If the same individual has a CACS of zero, then the risk becomes 3.5%. Importantly, the predictive value of CACS is incremental to that of traditional risk factors and risk calculators that have included CACS have outperformed established risk scores, such as the Framingham score and the 2013 American College of Cardiology (ACC)/American Heart Association (AHA) risk estimator (19). Today, CACS is an established way to better assess the Atherosclerotic Cardiovascular Disease (ASCVD) risk of asymptomatic individuals that otherwise fall into intermediate risk and start appropriate risk factor modification therapy (class IIb recommendation in European Society of Cardiology guidelines and IIa in ACC/AHA). The serial use of CACS is less clear, particularly in patients who are on statin therapy (20).

Nevertheless, the clinical application of CACS must take into account the pre-test probability of CAD, even when CACS is zero, as plaque can be non-calcified, particularly in younger, high-risk patients. Along these lines, clinical decisions for symptomatic patients should not be based solely on CACS, and in fact, CACS is not recommended in that scenario, as shown in the CORE64 trial where >10% of symptomatic patients with CACS of zero had obstructive CAD (21).

## Anatomic assessment

### Stenosis and plaque volume

Established applications of anatomic assessment *via* CCTA include the evaluation of symptomatic patients with suspected CAD. The degree of luminal stenosis as well as the presence of obstructive disease on CCTA, defined as >70% stenosis, correlate very well with mortality risk (22, 23). CCTA exhibits moderate to high sensitivity and specificity in discriminating lesion severity (24), with diagnostic accuracy curbed mainly by technical artifacts and limitations. Nevertheless, anatomic assessment with CCTA in the ISCHEMIA trial effectively ruled out left main disease, while clinical and stress testing were weak predictors (25). On the other hand, the advantage of CCTA compared to invasive angiography is the more precise evaluation of the presence and extent of non-obstructive lesions. This is clinically important since it has been shown that recurrent adverse events, specifically cardiovascular death, cardiac arrest, myocardial infarction, or unstable angina, arise from equally from culprit and non-culprit lesions during an index hospitalization (26). In fact, non-culprit lesions frequently cause mild stenoses. In contrast, severe plaque burden has been associated with adverse outcomes regardless of the degree of luminal stenosis (27). Of note, total plaque burden in asymptomatic individuals with type-2 diabetes has demonstrated additional prognostic value for cardiac events compared to clinical risk assessment and CACS (28). Coronary plaque burden on CCTA has also been shown to correlate with levels of high-sensitivity cardiac troponin T (hs-cTnT) in patients that were ruled out for myocardial infarction or even in asymptomatic patients. Even mild CAD has been associated with quantifiable circulating levels of hs-cTnT (29). As evidenced by *in vitro* studies, these elevated levels of hs-cTnT are not necessarily corresponding to myocardial necrosis, but they suggest that there is sufficient ischemia to result in cell stress, activation of caspase-3, cleavage, and release of cTnT (30). Possible mechanisms of ischemia are physical exercise, emotional stress, dislodgement of thrombi in coronary microvasculature and plaque erosion (31). CCTA-derived parameters can also be directly used for non-invasive evaluation of atherosclerotic features. For instance, positive remodeling of a plaque as well as non-calcified plaque volume have both been associated with increased risk for acute coronary syndromes (ACS) in a meta-analysis of 18 CCTA studies (32). Additional prognostic information could be obtained by serial examinations, as plaque progression has been associated with increased risk for ACS. However, serial CCTAs result in cumulative radiation exposure and are currently not recommended as standard of care. Studies are attempting to identify predictors of coronary plaque progression based on features of a baseline CCTA (33).

### High-risk plaques: Characteristics and prognostic value

The concept of high-risk or “vulnerable” plaques is that certain anatomic plaque characteristics can predict plaque rupture, erosion, and thus future thrombotic events. The concept originated from thin cap fibroatheromas, i.e., plaques with a necrotic or lipid core and a thin layer of overlying epithelial cells, which were associated with higher risk of adverse events (34). High-risk characteristics have been identified using CCTA include positive remodeling, low attenuation, spotty calcification, and the “napkin-ring” sign (35). Interestingly, these features respond well to statin use and may regress in serial scans (36). The greatest challenge in the application of these high-risk features is the complex natural history of coronary atherosclerosis itself; most plaque ruptures or erosions are now thought to be asymptomatic events that lead to plaque growth (37). In addition, it has been shown that coronary plaques have dynamic morphology and thin cap fibroatheromas may involve into thick cap fibroatheromas, i.e., lower risk plaques, at follow up (38). Equally importantly, symptomatic plaque rupture or erosion is determined by additional hemodynamic local factors such as endothelial shear stress, as well as systemic factors that determine the individual's thrombophilic state (38); thus prediction of clinically meaningful events is extremely complex and “vulnerable” plaques cannot be easily targeted for revascularization (10). Nevertheless, it is worth discussing the CCTA-derived characteristics of “vulnerable” plaques and their association with coronary events.

*Positive remodeling* is defined as the compensatory enlargement of the outer vessel wall at the site of an atherosclerotic lesion as the plaque burden increases (39). From a histopathological perspective, this enlargement results from macrophage infiltration and a large amount of necrotic core. CCTA can reliably measure and quantify positive remodeling using an index defined as the ratio of the vessel cross-sectional area at the site of maximal stenosis and the average of proximal and distal reference cross-sectional areas. A threshold of 1.1 is typically preferred in the assessment of CCTA images (40). Several studies have shown that a higher positive remodeling index can identify thin cap fibroatheroma (41, 42), as well as culprit lesions in ACS, but not in stable angina (43). In a prospective study including 74 participants with ACS or stable angina undergoing CCTA, positive remodeling was found in 96% of patients with ACS and ruptured fibrous caps, but only in 20% of those with ACS and intact fibrous caps and in 14% of individuals with stable angina (*p* <0.001) (44).

*Low attenuation* (<30 Hounsfield units) is typically used to easily describe plaques with a large lipid-rich necrotic core, as lipids typically have the lowest CCTA attenuation value. Ruptured plaques tend to have lower attenuation compared to stable lesions (44), while a lower plaque attenuation has been found in patients with ACS compared to those with stable angina (45). In a prospective study by Ozaki et al. including patients with ACS or stable angina, low attenuation plaques were more frequently observed in individuals with ACS and ruptured fibrous caps than in those with ACS and intact fibrous cap and those with stable angina (88, 40, and 18%, respectively; *p* = 0.001) (44). However, it is important to note that the CT attenuation value of a plaque may be influenced by various factors, including the concentration of the contrast agent, plaque burden, slice thickness, image noise, and tube voltage (46). Moreover, although fibrous tissue typically has higher attenuation values compared to lipids, there is a substantial overlap of densities often rendering their distinction from a CCTA impossible (47). Therefore, a reliable differentiation of lipid-rich and fibrous-rich plaques based on CCTA-attenuation remains challenging.

“*Napkin-ring” sign*. Refers to a specific plaque attenuation pattern with is a central area of low CCTA attenuation in contact with the arterial lumen surrounded by a ring-like higher attenuation plaque tissue (48). The central low attenuation corresponds to a large necrotic core, while the surrounding are of higher CCTA attenuation corresponds to fibrous plaque tissue, both of which are important predictors of plaque rupture (49). Napkin-ring sign is more frequent in thin cap fibroatheromas identified by optic coherence tomography (OCT) (41), and is strongly associated with future adverse cardiac events, independently from other high-risk plaque features (50). In a prospective study of 895 patients, the hazard ratio (HR) of ACS in patients with napkin-ring sign was 5.55 (*p* < 0.001) indicating a strong and statistically significant association (50). Although napkin ring signs seems to be a specific feature of rupture-prone plaques, its sensitivity remains relatively modest and thus, further in-depth analysis of plaque attenuation patterns is warranted.

*Spotty calcifications* are defined as <3 mm calcifications in plaques with a density of more than 130 Hounsfield units (51). Calcification marks local inflammation, which can exert mechanical effects on the plaque that make it susceptible to rupture (52). Spotty calcification has been associated with accelerated atherosclerosis progression in individuals with stable angina pectoris (53), while it has also been associated to culprit plaques in patients with ACS (54). In a study including 38 patients with ACS and 33 with stable angina pectoris, spotty calcification was significantly more frequent in ACS lesions (63 vs. 21%, *p* < 0.001), while large calcification was observed less frequently in ACS lesions than in stable angina (22 vs. 55%; *p* < 0.05) (43). An important limitation is that CCTA cannot visualize micro-calcifications that are smaller than 0.5 mm in diameter which are thought to be a common feature of unstable coronary lesions (55, 56).

## Hemodynamic assessment

### Fractional flow reserve

CCTA cannot reliably say whether a lesion is mild, moderate, or severe and thus, current research is focused on achieving a complementary functional assessment of coronary plaques that so far has been obtained by stress testing and invasive indices. If successful, it would tremendously improve the prognostic value of CCTA. Functional flow reserve (FFR) is defined as the ratio between the maximum achievable blood flow in the presence of coronary stenosis and the theoretical maximum flow if the stenosis was not present (57). FFR is measured invasively using pressure wires in the coronary arteries. Invasive FFR >0.75–0.8 indicates hemodynamically non-significant stenosis for which percutaneous coronary intervention can be deferred (58). Recent advantages have allowed a non-invasive calculation of FFR using CCTA and computational fluid dynamics. A negative CT-FFR, i.e., a value above a specific cut-off, carries the promise of safely deferring invasive angiography in patients with stable angina (59). However, CT-FFR has its limitations and introduces an error of its own, while an evidence-based cut-off value for non-invasive FFR is yet to be defined. In a small study of almost 190 patients with suspected CAD and intermediate coronary lesions, CT-FFR >0.8 was used to defer invasive angiography; patients had no adverse events occurring during a median follow-up of 12 months (60). In another study of around 250 patients that presented with acute chest pain and no known CAD, revascularization was deferred when CT-FFR was >0.8 with no difference in occurrence of major adverse cardiac events (61). Current evidence suggests that a dichotomous decision can be made for CT-FFR higher than 0.80 as well as for values equal or lower than 0.70, whereas for the range between 0.71 and 0.80 remains a “gray zone” and referral to invasive angiography should be considered individually (62).

### Myocardial perfusion

Another technique that can add functional information in CCT is myocardial computed tomography perfusion (CTP) (63). Moreover, dynamic CTP offers absolute quantification of myocardial blood flow, as in positron-emission tomography (PET) (64). Multicenter studies have demonstrated that myocardial CTP can provide incremental diagnostic value over CCTA alone for the identification of hemodynamically significant coronary artery disease (63, 65), similar to that of magnetic resonance perfusion (65). Additionally, dynamic CTP has been shown to be superior to machine learning empowered CT-FFR for identifying obstructive lesions (66). Dynamic myocardial CTP also has incremental predictive value over CCTA or clinical risk factors for the prediction of future major adverse cardiac events, allowing for improved risk stratification (67, 68). In one study, myocardial blood flow derived from dynamic CTP was the strongest predictor for major adverse cardiovascular events outperforming high risk plaque features and CT-FFR (69).

### Endothelial shear stress

Endothelial shear stress (ESS) is the frictional force produced when blood flows through an artery and has proven to trigger biological signaling in the endothelium (70). Physiologic ESS, typically found in straight vascular regions, upregulates anti-inflammatory genes and is atheroprotective. Low ESS, often found at branch points, bifurcations, and regions of high curvature, initiates cellular pathways that promote inflammation and are considered to be atherogenic (71). Specifically in native arteries, low ESS has been associated with the initiation and progression of atherosclerosis, development of high-risk plaque characteristics, need for revascularization, and major adverse cardiovascular events (26, 72). In stented arteries, low ESS has been correlated with neo-intima hyperplasia and neo-atherosclerosis, which can ultimately lead to further adverse cardiovascular events, including stent restenosis (73, 74). ESS is a promising hemodynamic index that can be evaluated using CCTA and computational fluid dynamics. As the lower imaging resolution of CCTA can have an impact on the accuracy of the estimated ESS, higher-resolution models reconstructed from a fusion of CCTA with intravascular imaging techniques, such as intravascular ultrasound (IVUS) and OCT, have been developed (75). Studies using CCTA-based models have shown that ESS assessment can provide incremental value in discriminating coronary segments more likely to exhibit atherosclerotic disease progression (76). In the Exploring the Mechanism of Plaque Rupture in Acute Coronary Syndrome Using Coronary CT Angiography and Computational Fluid Dynamics (EMERALD) study including 72 patients with ACS that had previously underwent CCTA, hemodynamic assessment, including ESS evaluation, provided additional value in identification of high-risk plaques that had ultimately caused ACS (72). However, further information on the clinical utility of CCTA-ESS remains to be seen.

## Perivascular adipose tissue

Recent evidence suggests that perivascular adipose tissue (PVAT) is linked to atherosclerosis as a key regulator and sensor of coronary inflammation (77, 78). PVAT lies in proximity with the vascular wall and plays an important role in the pathogenic process of atherosclerosis by regulating the local microenvironment through the release of a variety of substances, such as bioactive adipokines, cytokines, and chemokines (77, 79). Pericoronary fat attenuation index (FAI) is a novel CCTA-derived biomarker based on the concept that spatial changes in composition induced by inflammation cause a shift in CT attenuation toward more negative HU values (80). FAI is increased in patients with CAD compared to healthy individuals and is particularly increased around culprit lesions of patients presenting with ACS (80). In the Cardiovascular Risk Prediction using Computed Tomography (CRISP-CT) study, two independent cohorts with a total of 3,912 participants undergoing CCT were used to derive and validate the prognostic value of perivascular fat attenuation mapping (12). Based on the results, higher FAI values around proximal right coronary artery and left anterior descending artery were also associated with a higher risk for all-cause death and cardiac death (12). Finally, although FAI has been shown to be modifiable, as it decreased significantly when measured 5 weeks after an index event (80), whether risk-reduction therapies, such as statins, can reverse FAI is yet to be investigated. Future incorporation of CCT derived indices describing perivascular adipose tissue inflammation may improve risk assessment in patients with CAD (Table 1).

**Table 1:** Cardiac computed tomography derived parameters: pros, cons, and clinical value.

**Parameters**	**Pros**	**Cons**	**Clinical value**
CACS	- Low radiation - No contrast - Quick - Inexpensive - Reproducible	- Unclear value of serial CCT assessments - Must consider pre-test probability of CAD	- Good correlation with long-term risk of cardiac events - Incremental predictive value on top of traditional risk factors
**Anatomic assessment**			
Stenosis and plaque volume	- Precise evaluation of presence and extent of non-obstructive lesions	- CCTA has moderate to high sensitivity and specificity in lesion severity	- Degree of stenosis correlates well with mortality risk - Severe plaque burden correlates with adverse cardiac outcomes - Plaque progression on serial CCTAs correlates with risk of ACS
High-risk plaque features		- Dynamic morphology of plaques not captured - Need to consider additional thrombophilic factors	- Predict plaque rupture/ erosion - Respond well to statin use
*Positive remodeling*			- Correlates with TCFA and culprit lesions in ACS
*Low attenuation*		- Influenced by contrast concentration, plaque burden, slice thickness, image noise, tube voltage - Challenging distinction of lipid vs. fibrous-rich plaques	-Lower attenuation in ruptured plaques and in ACS compared to stable lesions and stable angina
*Napkin-ring sign*	- Good specificity	- Modest sensitivity	- Correlates with TCFA and future cardiac events
*Spotty calcification*		- Micro-calcifications cannot be visualized with CCTA	- Correlates with accelerated CAD progression and culprit plaques in ACS
**Hemodynamic assessment**			
FFR	-Functional assessment of lesion	- Gray zone; No evidence-based cut-off value	- FFR > 0.75–0.8 indicates hemodynamically significant stenosis - Negative CT-FFR can safely defer invasive angiography
CTP	- Identification of of myocardial perfusion defects - Detection of hemodynamically significant stenosis		- Absolute quantification of myocardial blood flow similar to PET - Incremental diagnostic value over CCTA alone and CT-FFR for the identification of hemodynamically significant CAD - Incremental predictive value over CCTA, CT-FFR, or clinical risk factors for the prediction of future major adverse cardiac events
ESS		- Lower accuracy (except if CCTA is fused with intracoronary imaging techniques)	- In native arteries: associated with initiation and progression of atherosclerosis, development of high-risk plaques, need for revascularization, and major adverse events - In stented arteries: associated with neo-intima hyperplasia and neo-atherosclerosis
PVAT		- No data available regarding risk-reduction therapies (e.g., statins)	- Higher FAI associated with: ° CAD ° ACS culprit lesions ° All-cause mortality ° Cardiac mortality

*CACS, Coronary artery calcium score; CCT, Cardiac computed tomography; CAD, Coronary artery disease; CCTA, Cardiac computed tomography angiography; ACS, Acute coronary syndromes; TCFA, thin cap fibroatheroma; FFR, Fractional flow reserve; CT-FFR, Computed tomography-FFR; CTP, Computed tomography perfusion; PET, Positron emission tomography; ESS, Endothelial shear stress; PVAT, Perivascular adipose tissue; FAI, Fat attenuation index*.

## Current limitations

Despite its many strengths, CCT for the evaluation of CAD has a few limitations. First, a variety of factors can introduce noise, therefore limiting image quality. Examples include increased heart rate, arrhythmias, high-density materials (e.g., calcium, stents), high body mass index, and poor patient cooperation (e.g., movement, inappropriate breath control). It is typical for calcifications to appear falsely enlarged due to blooming or partial volume artifacts and thus, result in overestimation of the extent of CAD (81). Studies have shown that CCT may provide conflicting results with overestimation or underestimation of the lumen area when compared to IVUS (82, 83). For these reasons, invasive coronary angiography remains the gold standard for coronary lesions and in many cases, CCT can only be used as a gatekeeper to more invasive testing. Additionally, different scanners, protocols, and technical parameters can lead to a variation of results and false interpretations (84). Regarding the use of CCT, another important concern is exposure to radiation and the risk of cancer (85). Furthermore, the contrast required for CCTA carries the risk of contrast-induced nephropathy (86). Finally, as CCT and CCTA are primarily anatomic modalities that assess coronary stenosis, plaque burden and characteristics, the addition of a functional test to increase diagnostic accuracy is often clinically useful (87, 88). In other words, a multimodality or hybrid imaging approach to CAD can be pursued in the appropriate clinical setting.

## Cardiac computed tomography and “omics”: Radiomics, proteomics, and lipidomics

The development of radiomics, as well as the integration of proteomics and lipidomics with CCT images, show promise for increased diagnostic and prognostic performance of CCT images, yielding potentially important clinical benefits. In fact, a radiomic-based machine learning model to identify advanced atheromatous lesions was found to be superior when compared to visual assessment (89). Similarly, in the CRISP-CT study, radiomic mapping of the perivascular fat was shown to offer incremental value for predicting adverse cardiac events compared to traditional risk profile assessment or presence of high-risk plaque features (90). Additionally, several studies have screened the proteome aiming to investigate whether specific proteins can relate to the presence or extent of coronary atherosclerosis. To illustrate, in a CCTA-based cohort, four proteins involved in vascular processes were found to be specifically associated with either low or high CAD burden (91). Similarly, other studies have assessed the association between lipid profiles with atherosclerotic plaque findings (92). In general, the new era of proteomics and lipidomics could provide a deeper insight to the atherosclerotic process beyond traditional risk factors as well as identify potential and novel treatment targets promising a more individualized patient care in the future.

## Dual-energy and photon-counting computed tomography

In the past few years, new CCT techniques such as dual-energy CCT and photon-counting CCT have aimed at improving the spatial resolution and the contrast-to-noise ratio of CT scanners (93). Dual-energy CCT uses two CCT datasets acquired with different photon spectra and tube potentials. With regards to CAD, it can enhance luminal assessment, evaluation of atherosclerotic plaque composition and evaluation of myocardial perfusion (94). Future studies are still needed to ensure wide external validation and existence of incremental clinical value when compared to already established technology. Photon-counting CCT allow for direct detection of incident X-ray photons, in contrast to energy-integrating scanners (i.e., those currently used in clinical practice), which are based on light photon detectors that are converted to electric signals at scintillation layer (95). This allows for increased signal to noise ratio, which in turn allows for better image quality, decreased radiation doses and elimination of beam hardening artifacts. In addition, photon-counting CCT could enhance the ability of the CCT scanner to detect different combinations of contrast agents, such as atherosclerotic plaque-specific nanoparticles (96). Although photon-counting CCT holds a great promise for the future, its access is currently limited to a few centers, and many technical challenges still need to be overcome prior to wide implementation.

## Conclusion and future perspectives

CCT has become more widely available and is currently viewed as a first-line diagnostic test CAD. In certain asymptomatic patients, it also provides improved risk stratification, guiding prevention of future adverse events. Further research on image processing could bridge the diagnostic accuracy gap between invasive and non-invasive coronary angiography in regards to grading of calcified plaques and hemodynamic evaluation of lesions. Evaluation of pericoronary fat inflammation could also be broadly adopted for the prediction of future adverse events. In addition, technologic developments aiming at reducing radiation (90) could result in the expansion of CCTA and help clarify the role of serial scanning. New techniques, such as dual-energy CCT and photon-counting CCT also hold promise at achieving higher spatial resolution and improving contrast-to-noise ratio. Finally, incorporating CCT and CCTA with machine learning and computational fluid dynamics could lead to more precise and individualized risk assessment.

## Author contributions

All authors contributed to the writing and revision of the manuscript and approved the final manuscript.

## Funding

MINOCA-GR study (Role of CCT in the diagnostic evaluation and risk stratification of patients with myocardial infarction and non-obstructive coronary arteries), an investigator-initiated study, supported by Menarini Hellas S.A (Ref. No. 27.11.2020/72059).

## Conflict of interest

The authors declare that the research was conducted in the absence of any commercial or financial relationships that could be construed as a potential conflict of interest.

## Publisher's note

All claims expressed in this article are solely those of the authors and do not necessarily represent those of their affiliated organizations, or those of the publisher, the editors and the reviewers. Any product that may be evaluated in this article, or claim that may be made by its manufacturer, is not guaranteed or endorsed by the publisher.
